# Sarcoma IL-12 overexpression facilitates NK cell immunomodulation

**DOI:** 10.1038/s41598-021-87700-2

**Published:** 2021-04-15

**Authors:** Mary Jo Rademacher, Anahi Cruz, Mary Faber, Robyn A. A. Oldham, Dandan Wang, Jeffrey A. Medin, Nathan J. Schloemer

**Affiliations:** 1 Department of Pediatrics, Medical College of Wisconsin, Milwaukee, WI USA; 2 Department of Biochemisty, Medical College of Wisconsin, Milwaukee, WI USA; 3 Department of Medical Biophysics, University of Toronto, Toronto, ON Canada; 4 Department of Microbiology and Immunology, Medical College of Wisconsin, Milwaukee, WI USA; 5 Laboratory of Molecular Immunology and Immunotherapy, Blood Research Institute, Versiti, Milwaukee, WI USA

**Keywords:** Oncology, Bone cancer, Cancer therapy, Paediatric cancer, Sarcoma, Translational immunology, NK cells

## Abstract

Interleukin-12 (IL-12) is an inflammatory cytokine that has demonstrated efficacy for cancer immunotherapy, but systemic administration has detrimental toxicities. Lentiviral transduction eliciting IL-12-producing human sarcoma for autologous reintroduction provides localized delivery for both innate and adaptive immune response augmentation. Sarcoma cell lines and primary human sarcoma samples were transduced with recombinant lentivirus engineering expression of human IL-12 (hu-IL-12). IL-12 expressing sarcomas were assessed in vitro and in vivo following implantation into humanized NSG and transgenic human IL-15 expressing (NSG.Tg(Hu-IL-15)) murine models. Lentiviral transduction (LV/hu-IL-12) of human osteosarcoma, Ewing sarcoma and rhabdomyosarcoma cell lines, as well as low-passage primary human sarcomas, engendered high-level expression of hu-IL-12. Hu-IL-12 demonstrated functional viability, eliciting specific NK cell-mediated interferon-γ (IFN-γ) release and cytotoxic growth restriction of spheroids in vitro. In orthotopic xenograft murine models, the LV/hu-IL-12 transduced human sarcoma produced detectable IL-12 and elicited an IFN-γ inflammatory immune response specific to mature human NK reconstitution in the NSG.Tg(Hu-IL-15) model while restricting tumor growth. We conclude that LV/hu-IL-12 transduction of sarcoma elicits a specific immune reaction and the humanized NSG.Tg(Hu-IL-15) xenograft, with mature human NK cells, can define in vivo anti-tumor effects and systemic toxicities. IL-12 immunomodulation through autologous tumor transduction and reintroduction merits exploration for sarcoma treatment.

## Introduction

Modulating or harnessing the immune system through immunotherapy promises to treat a wide range of cancers. Despite some remarkable successes in hematological malignancies, this approach has not been efficacious in several classes of solid malignancies^1^. Bone and soft tissue sarcoma have demonstrated marginal clinical response to immunotherapy despite preclinical promise^2^. Improving the efficiency and potency of immune-based treatments, overcoming immune suppressive tumor microenvironments, and minimizing toxicities remain critical goals for treating sarcomas.

One strategy to augment the immune response is through the use of interleukin-12 (IL-12), a potent activator of both the innate and adaptive immune systems^3,4^. This cytokine stimulates both natural killer (NK) cells and T cells toward a proinflammatory, interferon-γ (IFN-γ) producing Type 1 differentiation response^5–8^. Co-activation of NK cells, in addition to T cells, provides a unique and potent alternative to current immunotherapy treatments that only modulate T cells. NK cells are innate immune cells capable of both direct tumor cytotoxicity and inflammatory cytokine production resulting in augmentation of the adaptive immune responses^9,10,11^. Sarcomas have been shown to display ligands recognized by NK cells^11–13^ and NK cell immune infiltration into solid tumors including sarcomas is correlated with improved outcomes^14,15^. This suggests that if NK cell responses to solid tumors can be augmented, then NK cell-mediated tumor clearance and a durable adaptive immune response can be elicited.

IL-12 has demonstrated potent anti-tumor effects in several systems, including solid malignancies and sarcomas, but has been limited by severe toxicities when administered systemically to humans^6,16–22^. Toxicities range from mild flu-like symptoms (fevers, chills, myalgias) and localized inflammation (colitis, hepatitis), to severe systemic inflammatory syndromes and even death. Strategies to harness the potent anti-cancer effects of IL-12 while limiting systemic toxicity have included direct tumor injection, membrane-bound delivery, and inducible expression^16,19,23^. Lentiviral expression in autologous tumor cells, administered as a vaccine, allows for limited systemic production while also activating the innate and adaptive immune systems outside of the immunosuppressive tumor microenvironment^24,25^. This mechanism has led to a phase I trial in acute myeloid leukemia (AML) (Clinical Trials Number: NCT02483312)^26^. Murine models have demonstrated the efficacy of autologous IL-12 tumor expression in murine solid malignancies including osteosarcoma^16^. Additionally, autologous transduction and reintroduction of human sarcoma for immunotherapy has demonstrated both feasibility and safety^27^. Those efforts and ongoing trials have centered on the adaptive immune response and GM-CSF, which has limited effects on augmentation of the NK cell population but more impact on activation/expansion of T-cells and myeloid precursors^28–30^. In this report, we demonstrate engineering of human sarcoma cell lines and primary tumors to express IL-12, and the subsequent human innate immune activation both in vitro and in vivo.

## Materials and methods

### Lentiviral (LV) constructs

LV/hu-IL-12: LV/hu-IL-12 was produced for this study by the Vector Production Facility at Medical College of Wisconsin(MCW) (headed by Dr. Medin). The lentiviral construct that engineers expression of a fused form of human IL-12 α chain (p35) and β chain (p40) was previously described^26^. The construct also contains the cDNA for a fusion of truncated low-affinity nerve growth factor receptor (ΔLNGFR) and thymidylate kinase (TMPK) following an internal ribosome entry site sequence. This allows for expression of a cell surface fusion, cell-fate control protein that can be used to identify/track vector transduced cells and also eliminate them by conversion of exogenously added azidothymidine (AZT) to a toxic metabolite should the need arise^30^. Prior DNA sequencing revealed two mutations in the IL-12 plasmid sequence which are not known polymorphisms and have since been corrected.

LV/enhanced green fluorescent protein (eGFP)/firefly luciferase (fLUC): LV/eGFP/fLUC was produced for this study by the Vector Production Facility at MCW. The lentiviral construct that engineers expression of both eGFP and fLUC was previously described^31^.

### Use of human participant materials and experimental animals

Collection and utilization of primary human sarcoma samples was approved by and performed in accordance with the Children’s Wisconsin (CW) Institutional Review Board (IRB) #1. Approval ID is CW IRB (FWA00001809) Board #1 (Registration# IRB00002082) and Board #2 (Registration# IRB00006080). Participants or their legal designees/guardians consented to participate under informed consent documents within Human studies IRB Approval:1347982. Samples were collected from residual tissue of a clinically indicated procedure. No patient procedures were performed exclusively for research purposes. Collection and utilization of primary human NK cells was approved by and performed in accordance with the MCW IRB. Approval ID is Blood Center of Wisconsin which has a Federal Wide Assurance (FWA00005505) and cedes IRB review to the MCW IRB Committee #1 (Registration# IRB00001395). NK cells obtained under the human studies IRB approval: 00031019. For this study experimental animal utilization was approved by and performed in accordance with the MCW’s Institutional Animal Care and Use Committee (IACUC). This institution has an Animal Welfare Assurance on file with the Office of Laboratory Animal Welfare. The Assurance Number is D16-00064 (A3102-01). MCW IACUC assigned approval: 00006615.

### Cell culture

143B, KHOS-240S, A673, and RD cells were purchased from ATCC (Manassas, VA) and maintained in their ATCC-recommended medium containing fetal bovine serum (FBS) (Fisher)^32–34^. The NK-92mi cell line was purchased from ATCC (Manassas, VA) and cultured in Lonza X-VIVO 20 media (Fisher) supplemented with 5% GemCell Human Serum AB (Gemini Bio-Products). NK-92mi does not require exogenous IL-2 administration^35^. Cell lines were periodically tested to exclude mycoplasma contamination.

Human primary sarcoma samples obtained through the human subjects research-approved CW IRB protocol #1347982, as detailed above, were cultured in Dulbecco’s Modified Eagle’s Media (DMEM) (Gibco by Thermo Fisher Scientific) supplemented with 10% FBS and 1 × PSQ (2 mmol/l glutamine, 100 U/mL penicillin, and 100 mg/mL streptomycin, Gibco by Thermo Fisher Scientific). All cells were maintained in a humidified incubator at 37 °C with 5% CO_2_.

Human primary NK cells were obtained through human subjects research-approved MCW IRB protocol #00031019, as detailed above, and collected from a whole blood sample. A Lymphoprep (Stem Cell, Cat#07801) gradient was utilized according to the manufacturer’s instructions for peripheral blood mononuclear cell (PBMC) isolation. PBMC product was washed with PBS and subjected to Human CD56 positive selection kit (Stem Cell, Cat#17855) to purify NK cells. Isolated primary human NK cells were then resuspended at 1 × 10^6 cell/mL with 500 U/mL of IL-2 for 30 min prior to assay utilization.

### Transduction of sarcoma cells

Sarcoma cell lines (143B, KHOS-240S, A673, and RD) and primary samples were transduced by LV/hu-IL-12 at an estimated multiplicity of infection (MOI) of 2. During the transductions, cells were incubated overnight in a humidified incubator at 37 °C with 5% CO_2_, then washed and resuspended in fresh culture medium the next morning. Transgene expression was determined by flow cytometry (FCM) assessment of LNGFR and subsequent measurement of the concentration of IL-12 in the supernatants with a commercially available hu-IL-12 ELISA kit (Invitrogen by Thermo Fisher Scientific).

### Spheroid formation and growth

LV-transduced and non-transduced cells were washed twice and 1000 cells at 1000 cells/100 µL, unless stated otherwise in the figure legend, were seeded in a 96-well Nunclon Sphera ultralow-affinity plate and cultured in their ATCC-recommended media to allow for spheroid formation verified by ZOE fluorescent Cell Imager (Bio-Rad). NK-92mi were labeled with Cell Trace Far-Red (Thermo Fisher Scientific) and 1000 cells at 1000 cells/100 µL, unless stated otherwise in figure legend, were added to each well after 48 h. Daily visual confirmation of spheroid formation, measurement of diameter using ImageJ software^36^, and NK-92mi infiltration were documented via a ZOE fluorescent Cell Imager (Bio-Rad) recorded for 96 h.

### ^51^Chromium (^51^Cr) cytotoxicity

Spheroid: LV-transduced and non-transduced cells were washed twice and loaded with ^51^Cr for 2 h, washed, and 1000 cells seeded in a 96-well Nunclon Sphera plate, ultralow-affinity, at a concentration of 1000 cells/200 µL and cultured in their ATCC-recommended media to allow for spheroid formation. After 24 h, NK-92mi-mediated cytotoxicity against 143B, A673, and RD spheroids was quantified using ^51^Cr-release assays at varied effector:target ratios (E:T ratio) as described^37^.

Monolayer: LV-transduced and non-transduced cells were washed twice and loaded with ^51^Cr for 2 h, washed, and 5000 cells seeded in a 96-well plate, at a concentration of 5000 cells/100 µL with or without 0.5 mg/mL anti-IL-12 blocking antibody (R&D #MAB1510)^38^. NK-92mi were added immediately at varied E:T ratios and cytotoxicity quantified at 4 h. NK-92mi percent specific lysis was calculated using equation: Percent cytotoxicity = (experimental ^51^Cr-release/absolute ^51^Cr-release)*100.

### Cell fate control

LV-transduced and non-transduced cells were washed twice and 1000 cells at a concentration of 1000 cells/200 µL were seeded in a 96-well Nunclon Sphera plate, ultralow-affinity, in their ATCC-recommended media to allow for spheroid formation. After 24 h, AZT was added at 0, 10, 100, and 1000 µmol/L for up to 5 days. Media and AZT were refreshed every 2 days in the cell culture. Daily visual confirmation of spheroids and measurement of diameter recorded for 96 h were performed using a ZOE fluorescent Cell Imager (Bio-Rad) and using ImageJ software^36^.

### Cytokine assessments

IL-12: To measure levels of human IL-12 p70, cells were washed twice and 1000 cells were seeded in a 96-well Nunclon Sphera plate, ultralow-affinity, at a concentration of 1000 cells/200 µL and cultured in their ATCC-recommended media to allow for spheroid formation. The cell culture supernatant was then collected and the concentration of human IL-12 p70 was measured with a hu-IL-12 ELISA kit (Invitrogen by Thermo Fisher Scientific) according to manufacturer’s instructions.

IFN-γ: To measure levels of human IFN-γ produced by NK-92mi, they were added to 48-h established spheroids at a ratio of 10:1, unless otherwise stated in figure legends, with supernatant collection after 24 h of co-culture. For conditioned media only activations we plated 100,000 NK-92mi at a density of 100,000 cells/100 µL and exposed to 100 uL of conditioned media from sarcoma spheroids, with supernatant collected after 4 h. Primary human NK cells were collected as above and 100,000 cells were added at 100,000 cells/100 µL to sarcoma cells in ratios per figure legends or exposed to 100uL of conditioned media from sarcoma spheroids. Supernatant was collected after 6 h. The secreted IFN-γ was measured with a human IFN-γ ELISA kit (Invitrogen by Thermo Fisher Scientific) according to manufacturer’s instructions. Anti-IL-12 blocking antibody (R&D #MAB1510) was utilized at a concentration of 0.5 mg/mL^38^.

Premixed multiplex human magnetic Luminex Assays (R&D systems): To measure levels of human cytokines in the humanized murine xenograft models. Serum was collected as described below and frozen at -20 degrees Celsius for batched assessment according to manufacturer’s instructions (Cat# LXSAHM-08, Lot# L137952).

### Antibodies

The following antibodies were utilized: CD271 Alexa Fluor 647 anti-human (BD Biosciences #560326), CD45 APC anti-mouse (BioLegend #147708), CD45 Pacific Blue anti-human (BioLegend #304029), NKp46 PE anti-human (BioLegend #331908), CD16 APC anti-human (BioLegend #302012), CD56 Alexa Fluor 488 anti-human (BD Biosciences #557699), CD33 FITC anti-human (BioLegend #303304), CD19 PE-Texas Red anti-human (ThermoFisher #MHCD1917), CD3 PE-Cy7 anti-human (Cell Signaling #62670S), CD8a APC anti-human (BioLegend #300912), CD8 PE anti-human (Thermo Fisher #12-0089-42), CD4 Alexa Fluor 488 anti-human (BioLegend #317420), CD4 PE-Texas Red anti-human (Thermo Fisher #MHCD0417), CD4 Pacific Blue anti-human (Thermo Fisher #MHCD0428), IL-12 anti-human (R&D #MAB1510).

### Flow cytometry assessment

Single-cell suspensions were prepared and stained with fluorescent-labeled monoclonal antibodies in 1% FCS-PBS as previously described^39^. Standard FCM analyses were performed on an LSR-II (BD) and analyzed by FlowJo (Ashland, OR).

### Murine humanization

All mice were maintained in pathogen-free conditions at the Biological Resource Center at MCW. NSG and NSG.Tg(Hu-IL-15) mice (Stock No. 005557: NOD.Cg-Prkdc^scid^ Il2rg^tm1Wjl^/SzJ) were obtained from Jackson Laboratory (Bar Harbor, ME). Female and male mice were used at the ages of 6 to 12 weeks for lab humanization. All animal protocols and human CD34 cell usage were approved by the respective institutional MCW IACUC and CW IRB committees. Animals were irradiated with 240 cGy using a Gammacell-40. Following that, they received 8 × 10^5^ of G-CSF mobilized peripheral blood human CD34 + cells (StemCell Technologies, Cat # 70060.3, Lot #1705230096) via tail vein injections. Enrofloxacin prophylaxis was administered in drinking water at 0.1 mg/mL for 4 weeks. PBMCs were collected and, following red blood cell lysis (Thermo Fisher Scientific), were analyzed by FCM for immune cellular reconstitution as above. Commercially humanized mice obtained from Jackson Laboratory (NSG.Tg(Hu-IL-15)) were used at 12–20 weeks with age matched NSG also obtained from Jackson Laboratory (Bar Harbor, ME).

### Sarcoma adoptive transfer model

A673 (human Ewing sarcoma), 500,000 cells in 100 µL of PBS, were injected in the right quadriceps femoris muscle. The mouse weights and tumor measurements were serially recorded. Tumor volume calculated with the following equation Volume = (length*width^2)/2. Animals were euthanized for weight loss of 20%, tumor dimension of > 20 mm or clinical disability or ambulation limitations per AUA. At euthanasia necropsy was performed and tumors collected for histologic assessment. Mice were bled at regular intervals from their submandibular vein or terminal cardiac puncture. Approximately 100 µL of blood was collected and spun at 300 × *g* for 4 min. Serum was collected for cytokine analysis and analyzed as above with hu-IL-12 and human IFN-γ ELISA kits (Invitrogen by Thermo Fisher Scientific) or premixed multiplex human magnetic Luminex assay (R&D Systems) according to manufacturer’s instructions.

### Statistical analyses

Statistical analyses were performed using unpaired, two-tailed Student’s t-tests for comparison between two sets of quantitative data. A repeated measures two-way ANOVA with mixed effects modeling to account for deceased animals in comparisons of three or more groups was performed for tumor volume and mouse weight comparisons in in vivo tumor models, a Geisser-greenhouse correction was utilized for lack of sphericity. A Log-rank (Mantel-Cox) test was utilized for survival curve comparison. Further statistical tests are described in figure legends. P values of ≤ 0.05 were considered statistically significant; Non-significant, *p* > 0.05. *, *p* < 0.05; **, *p* < 0.001; ***, *p* < 0.0001. Statistical analyses were performed using GraphPad Prism version 9.0.0 for Windows, GraphPad Software, San Diego, California USA, www.graphpad.com.

## Results

### LV/hu-IL-12 transduction correlates to IL-12 production in human sarcoma

IL-12 is comprised of a 35 kDa light chain and a 40 kDa heavy chain forming a heterodimer that is produced primarily by dendritic cells. For autologous expression of hu-IL-12, the p35 and p40 subunits are joined by an elastin linker sequence forming the p70 heterodimer^26,40^. Further, the lentivector we employed utilized a ΔLNGFR for transduction efficiency assessment and an engineered TMPK/AZT cell-fate control system^41,42^.

**Figure 1:**
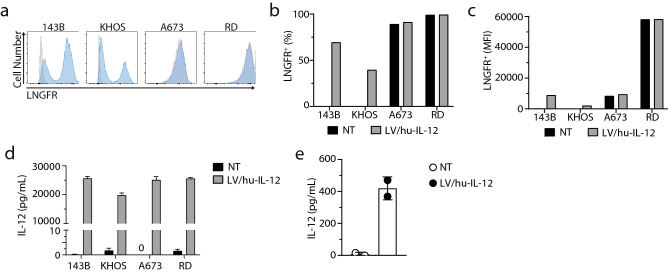
Human sarcoma lines for osteosarcoma (143B and KHOS-240S), Ewing sarcoma (A673), and rhabdomyosarcoma (RD) were transduced with LV/hu-IL-12 containing ΔLNGFR for surface tracking and cell-fate control. (**a**) LNGFR on the surface of the cell was measured by FCM > 72 h after transduction. (**b**,**c**) Surface LNGFR expression was quantified by percent positivity and mean fluorescent intensity (MFI). (**d**) The IL-12 concentration in the supernatant of transduced or non-transduced cells after 4-h incubation, measured by ELISA. Data represents 3 replicates measured in triplicate, displayed mean ± standard deviation. (**e**) Primary human sarcoma patient sample transduced with LV/hu-IL-12. Supernatant was collected 18 h after media change, and an IL-12 ELISA was performed. Individual transductions are displayed with mean ± standard deviation.

Osteosarcoma, Ewing sarcoma, and rhabdomyosarcoma cell lines were utilized to demonstrate feasibility across a variety of sarcomas. Transduction efficiency was assessed via FCM for ΔLNGFR expression (Fig. 1a). This read-out demonstrated the successful transduction of osteosarcoma (143B and KHOS-240S) cells. For Ewing sarcoma (A673) and rhabdomyosarcoma (RD) cells, FCM for ΔLNGFR expression was unable to differentiate transduced versus control cells due to endogenous LNGFR expression (Fig. 1b,c). These sarcomas were expanded without clonal selection to form single well spheroids. The specific production of IL-12 in the supernatant of transduced cell spheroids verified the successful LV transduction (Fig. 1d). Importantly, the transduction did not significantly alter the growth of tumor spheroids (Supplementary Figure S1). Additionally, the efficacy of the TMPK cell-fate control system was verified in the presence of increasing concentrations of AZT (Supplementary Figure S2a and S2b). Primary human sarcomas obtained and maintained in low passage culture were also transduced under the same conditions and demonstrated selective IL-12 secretion by ELISA (Fig. 1e).

### LV/hu-IL-12 transduction induces NK cell-mediated IFN-γ production

**Figure 2:**
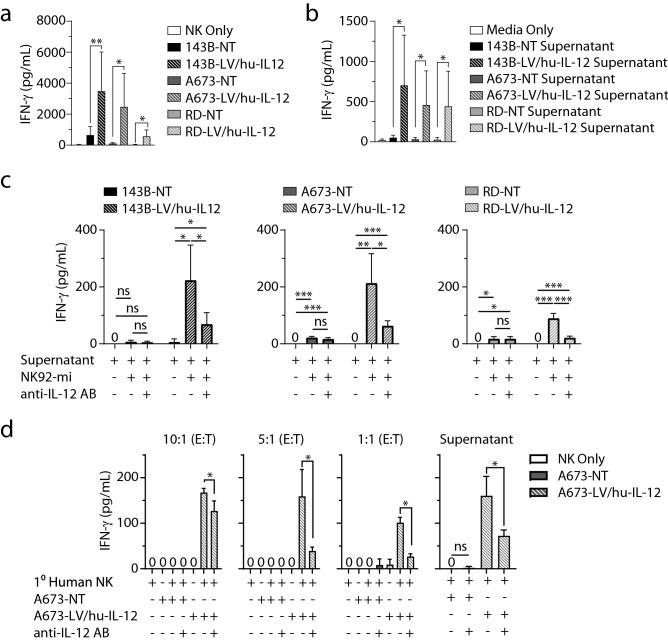
(**a**) Non-transduced (NT) and LV/hu-IL-12 transduced human sarcoma lines for osteosarcoma (143B), Ewing sarcoma (A673), and rhabdomyosarcoma (RD) were plated in ultralow adherent 96-well plates. After 48 h of growth, media or NK-92mi at a 10:1 ratio were added. Supernatants were assessed by ELISA for IFN-γ. N = 12; 3 independent experiments, displayed mean ± standard deviation. (**b**) Non-transduced and LV/hu-IL-12 transduced human sarcoma lines were plated. 4 h later conditioned supernatant was collected and 100 µL applied to NK-92mi cells. After an additional 4 h supernatant was collected, and IFN-γ was measured by ELISA. N = 6; 2 independent experiments, displayed mean ± standard deviation. (**c**) NK-92mi cells were incubated in the presence or absence of anti-IL-12 antibody and 100 µL of conditioned supernatant. After 4 h supernatant was collected, and IFN-γ was measured by ELISA. N = 6; 2 independent experiments, displayed mean ± standard deviation. (**d**) Primary human NK cells were plated with non-transduced (NT) and LV/hu-IL-12 transduced human Ewing sarcoma (A673) at the noted E:T ratios or conditioned supernatant (100 µL) in the presence or absence of anti-IL-12 antibody. After 6 h supernatant was collected, and IFN-γ was measured by ELISA. N = 3; displayed mean ± standard deviation. * = *p* < 0.05, ** = *p* < 0.001, *** = *p* < 0.0001.

Using NK cells, we next sought to determine if the linked heterodimer hu-IL-12 expressed by the transduced sarcomas was functional. We co-cultured sarcoma cells with NK-92mi cells and assessed for augmentation of interferon gamma (IFN-γ) production by ELISA. IFN-γ production by the NK-92mi cells was significantly augmented in wells with sarcomas transduced to express hu-IL-12 (Fig. 2a). This was demonstrated in osteosarcoma (143B) (*p* = 0.000971), Ewing sarcoma (A673) (*p* = 0.001010), and rhabdomyosarcoma (RD) (*p* = 0.000158) cells. There was not significant IFN-γ production in the absence of NK-92mi cells, absence of transduction, or transduction with a lentivector not coding for hu-IL-12 (*p* < 0.0001) (Supplementary Figure S3). NK-92mi were exposed to supernatants from sarcoma spheroid cultures to demonstrate that direct surface interaction was dispensable for activation in the setting of hu-IL-12 production. The NK-92mi production of IFN-γ was specifically stimulated by the use of supernatant from hu-IL-12 transduced osteosarcoma (143B) (*p* = 0.028675), Ewing sarcoma (A673) (*p* = 0.032811), and rhabdomyosarcoma (RD) (*p* = 0.042908) cells (Fig. 2b). Anti-human IL-12 neutralizing monoclonal antibody significantly reduced NK92-mi production of IFN-γ in response to supernatants from hu-IL-12 transduced osteosarcoma (143B) (*p* = 0.016455), Ewing sarcoma (A673) (*p* = 0.006253), and rhabdomyosarcoma (RD) (*p* = 0.000006) cells (Fig. 2c). Primary human NK cells were used to verify the specific IFN-γ production in response to non-transduced or hu-IL-12 transduced Ewing sarcoma in varied effector to target ratios (10:1, *p* = 0.000006; 5:1, *p* = 0.009510; and 1:1, *p* = 0.000125) and supernatant (*p* = 0.0029) (Fig. 2d). Anti-human IL-12 neutralizing antibody addition resulted in significant IFN-γ reduction in all ratios (10:1, *p* = 0.043222; 5:1, *p* = 0.025218; and 1:1, *p* = 0.000663) and supernatant (*p* = 0.0268) exposures from transduced sarcomas.

### NK-92mi-mediated sarcoma cytotoxicity augmented by human IL-12 overexpression

We then sought to determine the effect of human sarcoma IL-12 transduction on NK-mediated cytotoxicity. A three-dimensional tumor spheroid model was utilized since it has been shown to better replicate an in vivo tumor microenvironment with NK cell-mediated infiltration and activation/inhibition^43–45^. Hu-IL-12 transduced and non-transduced human sarcomas were grown for 48 h. NK-92mi cells were added and diameters of the sarcoma spheroids were tracked for 96 h. Previous reports have shown that infiltrative destruction of tumor spheroids takes significantly longer than monolayer tumor cultures^44^. Prior to administration, NK-92mi were labeled with CTFR allowing for specific tracking of NK-92mi cell infiltration into sarcoma spheroids.

**Figure 3:**
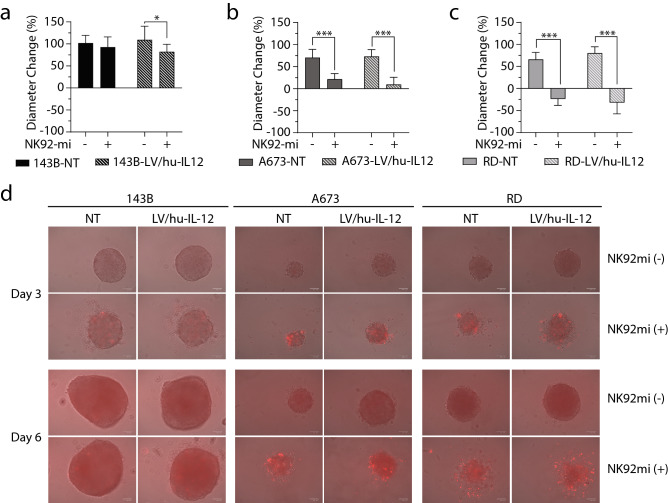
Non-transduced and LV/hu-IL-12 transduced human sarcoma lines for (**a**) osteosarcoma (143B), (**b**) Ewing sarcoma (A673), and (**c**) rhabdomyosarcoma (RD) were plated in ultralow adherent 96-well plates. After 48 h, spheroid formation was verified, diameters were recorded, and CTFR-labeled NK-92mi cells were added at a 1:1 ratio. Diameters were recorded every 24 h and displayed as Δ96 h. (**d**) Representative merged brightfield and red channel images. N = 8; 2 independent experiments, displayed mean ± standard deviation. * = *p* < 0.05, ** = *p* < 0.001, *** = *p* < 0.0001.

Osteosarcoma spheroids (143B) demonstrated no significant decrease in tumor diameter growth when NK-92mi cells were added to non-transduced sarcoma (*p* = 0.384067) and significant restriction only in the human IL-12 transduced (*p* = 0.049246) cells (Fig. 3a). In Ewing sarcoma (A673) (Fig. 3b) and rhabdomyosarcoma (RD) spheroids (Fig. 3c) there was a significant decrease in sarcoma spheroid growth when NK-92mi were added to both non-transduced (A673: *p* = 0.000028) (RD: *p* < 0.000001) and hu-IL-12 transduced cells (A673: *p* = 0.000001) (RD: *p* < 0.000001), with no significant differences between the presence or absence of IL-12 transduction. Representative images of diameter change and NK-92mi (CTFR +) infiltration are displayed (Fig. 3d). Confirmation of direct cytotoxicity without differences between presence or absence of IL-12 transduction was further demonstrated by a ^51^Cr assay with NK-92mi (Supplementary Figure S4a) and primary NK cells (Supplementary Figure S4b).

### Sarcoma spheroid size limits NK-92mi efficacy in growth restriction

**Figure 4:**
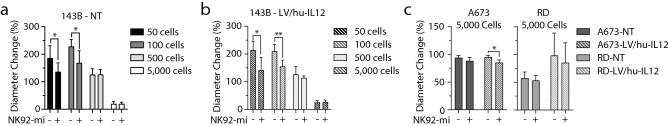
Osteosarcoma (143B) (**a**) Non-transduced (NT) and (**b**) LV/hu-IL-12 transduced sarcoma lines were plated in ultralow adherent 96-well plates at escalating doses from 50 to 5000 while (**c**) Ewing sarcoma (A673), and rhabdomyosarcoma (RD) plated at 5000 cells/well. After 48 h, spheroid formation was verified, diameters were recorded, and 1,000 NK-92mi cells were added. Diameters were recorded every 24 h and displayed as Δ96 h. N = 4, displayed mean ± standard deviation. * = *p* < 0.05, ** = *p* < 0.001, *** = *p* < 0.0001.

NK-92mi cells alone decreased the size of Ewing sarcoma and rhabdomyosarcoma spheroids without exogenous IL-12 (Fig. 3b,c). Conversely, in osteosarcoma, IL-12 overexpression was necessary to drive significant tumor reduction (Fig. 3a). We hypothesized that a more rapid rate of spheroid growth, and consequently a larger spheroid size at the time of NK-92mi addition, was responsible for this discrepancy. To test this hypothesis, we plated sarcoma cells in lower and higher quantities than were utilized in Fig. 3. After 48 h for spheroid formation, the NK-92mi cells were added and sarcoma spheroid size was tracked for 96 h. The smaller spheroids resultant from lower initial seeding numbers demonstrated significant decreases in growth in both the 143B non-transduced spheroids at 50 (*p* = 0.0237) and 100 cells (*p* = 0.0054) and also in the human IL-12 transduced 143B at 50 (*p* = 0.0045) and 100 cells (*p* = 0.0005) at initial seeding. The larger absolute initial seeding numbers yielded no significant differences in spheroid growth (Fig. 4a,b) (143B-NT: 500 (*p* = 0.9436) and 5,000 cells (*p* = 0.9776); 143B-LV/hu-IL-12:500 (*p* = 0.2460) and 5,000 cells (*p* = 0.7711)). Conversely, the significant differences seen in the A673 and RD sarcoma spheroids at an initial seeding of 1,000 cells (Fig. 3b,c) were abrogated with an increase to 5,000 cells at initial seeding (Fig. 4c). Hu-IL-12 expressing A673 (Ewing sarcoma) (*p* = 0.0016) was the exception, with retention of cytotoxicity augmentation—demonstrating the IL-12 benefit.

### Expression of IL-12 induces IFN-γ in vivo

**Figure 5:**
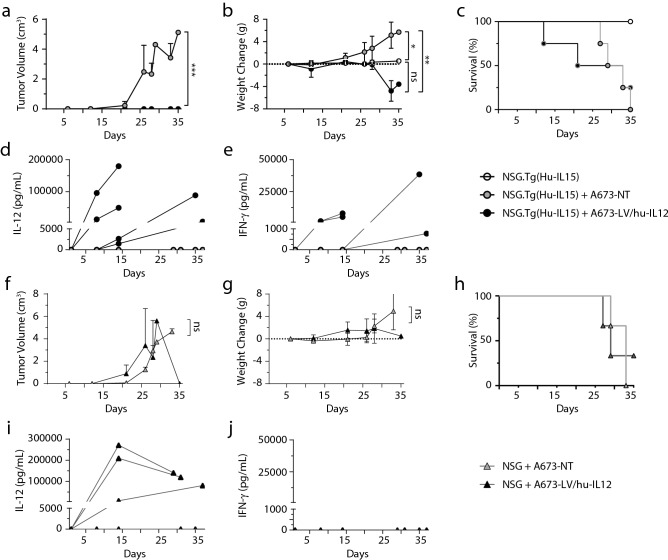
Ewing sarcoma (A673) non-transduced or LV/hu-IL-12 transduced sarcoma line cells were implanted in immunocompromised NSG and NSG-IL-15 transgenic mice with or without humanization. Tumor volumes (**a**,**f**) and weights (**b**,**g**) were serially recorded with daily survival/disability (**c**,**h**) assessments. Serum was collected from peripheral blood prior to injection and weekly until death or at 35 days post tumor injection. Multiplex cytokine analysis performed with display of serum IL-12 (**d**,**f**) and IFN-γ (**e**,**j**). N = 3–4, displayed as mean ± standard deviation. * = *p* < 0.05, ** = *p* < 0.001, *** = *p* < 0.0001.

To investigate if the effects seen in vitro would be translatable to an in vivo system, we tested a humanized NSG.Tg(Hu-IL-15) murine model maintaining a mature human NK population and non-humanized NSG murine models receiving either non-transduced or LV/hu-IL-12 transduced A673 (human Ewing sarcoma). Sarcoma cells were orthotopically implanted in the quadriceps femoris muscle. No statistical differences were observed in the degree of humanization between the NSG.Tg(Hu-IL-15) mice prior to receiving no sarcoma cells, non-transduced, and LV/hu-IL-12 transduced sarcoma cells (Supplementary Figures S5a–S5e). Murine tumors were monitored at least weekly, with mouse weights recorded and daily assessments made for disability. Peripheral blood was obtained prior to sarcoma implantation and weekly until stopping criteria were met. Surviving mice were euthanized at day 35 for histologic tumor assessment. The humanized NSG.Tg(Hu-IL-15) with LV/hu-IL-12 transduced cells had a significant reduction in tumor volume vs non-transduced cells (*p* = 0.0006) (Fig. 5a). However, there was no significant tumor volume difference in the NSG mice receiving the LV/hu-IL-12 transduced or non-transduced sarcoma cells (*p* = 0.4353) (Fig. 5f). NSG.Tg(Hu-IL-15) mice receiving non-transduced sarcoma cells demonstrated significant weight gain relative to mice who received LV/hu-IL-12 transduced sarcoma cells (*p* = 0.0011) and to mice receiving no sarcoma cells (*p* = 0.0338) (Fig. 5b). There was no significant difference in the NSG mice receiving the LV/hu-IL-12 transduced or non-transduced sarcoma cells (*p* = 0.4545) (Fig. 5g). There were no significant differences in survival between cohorts receiving LV/hu-IL-12 transduced and non-transduced sarcoma cells in either the NSG.Tg(Hu-IL-15) mice (*p* = 0.8962) (Fig. 5c) nor the NSG mice (*p* = 0.9709) (Fig. 5h). No mice in the NSG.Tg(Hu-IL-15) cohort receiving LV/hu-IL-12 transduced sarcoma met stopping criteria for tumor size; mice who met stopping criteria were for weight loss (n = 2) or post blood draw mortality (n = 1). All mice in the NSG.Tg(Hu-IL-15) cohort receiving non-transduced sarcoma met stopping criteria due to tumor size. All mice in both NSG cohorts met stopping criteria due to tumor size except one NSG mouse that received LV/hu-IL-12 transduced sarcoma that never demonstrated measurable IL-12, measurable tumor growth, or weight gain. On necropsy no mice had evidence of metastatic disease or cutaneous graft versus host disease. NSG.Tg(Hu-IL-15) mice receiving LV/hu-IL-12 transduced sarcomas developed measurable serum IL-12 with corresponding IFN-γ increases (Fig. 5d,e) while mice receiving non-transduced sarcoma developed no detectable amounts of either cytokine. NSG mice receiving LV/hu-IL-12 transduced sarcomas also demonstrated measurable serum IL-12 levels but without corresponding IFN-γ (Fig. 5i,j). Inflammatory and anti-inflammatory cytokine response was further characterized by the terminal NSG.Tg(Hu-IL-15) mice receiving hu-IL-12 sarcoma cells with significant increases of TNF-α (*p* = 0.000001) and IL-6 (*p* = 0.026431) with non-significant elevations in GM-CSF (*p* = 0.177808) and IL-10 (*p* = 0.102207) (Supplementary Figure S5f–S5i). No IL-1β or IL-2, which is produced primarily by T cells, was detected in any serum samples, supporting the primarily NK cell mediated inflammatory response^46,47^. Additional controls of sham transductions and NSG humanizations demonstrated no inflammatory responses (Supplementary Figure S6). In summary, in this transgenic model with endogenous human IL-15 supporting a primary human NK population, a human sarcoma producing hu-IL-12 following lentiviral transduction was able to elicit a specific inflammatory response with significant control of tumor growth.

## Discussion

Harnessing the combination of the innate and the adaptive immune system to safely treat cancer is the goal of immunotherapy. The majority of current treatments exclusively activate an adaptive immune response. However, the promise of the pro-inflammatory cytokine IL-12 is that it functions as a bridge activating both the innate and adaptive immune systems^7,8,20^.

Toxicity with systemic administration has limited IL-12’s clinical utility, but by local delivery at the cellular level through tumor cell transduction, autologous tumor reintroduction, and focused IL-12 expression, we hope to overcome this hurdle in solid malignancies^6,16^. This method has demonstrated safety in liquid malignancies (Clinical Trials Number: NCT02483312) and feasibility with other immune activating cytokines (Clinical Trials Number: NCT03495921). There are multiple other proposed methods under investigation for the safe utilization of IL-12 for anti-cancer therapy that may ultimately work well in concert with autologous reintroduction^23^.

There are several advantages to the schema of tumor transduction with IL-12 and autologous reintroduction that we are planning, including: (1) Localized: This cellularly localizes IL-12 with its gradient highest at the point where we would hope to elicit the targeted immune response of innate and adaptive immunity. (2) Avoids the tumor microenvironment: Autologous reintroduction of transduced cells elicits the immune responses outside of the native tumor immunosuppressive microenvironment. This is in contrast to intratumoral IL-12 injection or intratumoral introduction of viral vectors that permit for localized IL-12- mediated immune augmentation, but do so in the immunosuppressive tumor microenvironment. (3) No clonal expansion monitoring: IL-12 delivery via immune cells through co-transduction of IL-12 with chimeric antigen receptors (CARs) or specific T-cell receptors (TCRs) requires years of immune monitoring for clonal expansion of the administered product or, in the case of non-autologous products lymphodepleting chemotherapy, for tolerance. By administering an autologous tumor product that will be cleared with the desired immune response, the clonal monitoring requirement is eliminated. Further, the inclusion of a cell fate control or “suicide system” provides additional safety with the reintroduction of a transduced sample that can be readily cleared should the need arise (Supplementary Figure S2). Of note, the number of transduced cells reintroduced to patients is several orders of magnitude lower than the number of existing or native cancer cells in a single 1 cm tumor remaining in patients^48^. (4) Low lentiviral utilization: Since not every cell autologously administered needs to be hu-IL-12 producing, this is permissive for low MOI, transduction heterogeneity, and reduced vector utilization^16,26,40^. This limits potential genotoxicity and is critical for conserving good manufacturing practice grade lentivector, a limited and expensive reagent. (5) Multiple antigen exposure: The activation of the patient’s sarcoma and subsequent immune stimulation is not limited to a single antigen display, as is the case for standard CAR or TCR-based therapy. Instead, the immune system sorts out the dominant epitopes itself; this can even lead to protection from tumor re-challenge at a much later date^39^.

In our study we successfully demonstrate that human IL-12 can be expressed in human sarcoma cell lines derived from osteosarcoma, Ewing sarcoma, and rhabdomyosarcoma, as well as in primary sarcoma samples following lentiviral transduction (Fig. 1). The hu-IL-12 is functional with regard to the NK cell activation and can elicit a specific IFN-γ production response (Fig. 2). Sarcoma transduction with LV/hu-IL-12 aims to elicit an innate immune response and ultimately drive specific tumor cytotoxicity, not only in the transduced cells but also in non-transduced sarcoma as well. This would imply an innate and adaptive immune response. Importantly, the LV/hu-IL-12 was not required for NK cell-mediated cytotoxicity (Figs. 3, 4 and Supplementary Figure S4). In vitro LV/hu-IL-12 transduction led to limited augmention of the cytotoxic response (Figs. 3a and 4c) supporting the idea that activation of multiple immune components will be necessary for fully effective sarcoma therapy^49^. Further, the size of the sarcoma limited the direct NK cell-mediated cytotoxicity regardless of transduction status (Fig. 4). This illustrates the continued need to combine novel immunotherapy treatments with conventional chemotherapy and radiation for sarcoma cytoreduction and ultimately eradication^50^.

Our in vivo model utilized the humanized transgenic NSG expressing hu-IL-15 with a mature NK cell population (Fig. 5a–e) and demonstrated a significant restriction of tumor growth only with the introduction of the LV/hu-IL-12 transduced sarcoma (Fig. 5). We detected IL-12 systemically following tumor inoculation and a corresponding inflammatory response (Fig. 5 and Supplementary Figure S5). This systemic inflammatory detection, whilst necessary for the current model to demonstrate the in vivo activation of NK cells is likely the cause of the weight loss we observed and could be regulated in subsequent study. There are several IL-12 expression systems currently under investigation with either transmembrane anchored or molecular gene activated expression that attempt to minimize systemic release of IL-12 and consequently systemic toxicity^23^. Alternatively, the immune activation can be achieved with a smaller transduced cell dose^16^ or outgrowth of the autologous reintroduced cells limited by utilization of the cell fate control system or pre-delivery irradiation. Humanized murine models maintaining NK cells are necessary to study these systemic toxicities and efficacy. Avenue for future study will include the effect of localized IL-12 on the tumor microenvironment and tumor infiltrating lymphocytes as well evaluation of the durability of the immune response with tumor rechallenge models.

## Conclusion

IL-12 overexpression in autologous tumor cells is a viable method of immunomodulation that merits exploration for the treatment of sarcoma. IL-12 activates NK cells, which have a known role in sarcoma outcomes and are a key bridge between the adaptive and innate immune systems. We report that overexpression of hu-IL-12 in human sarcomas elicits a specific immune response both in vitro and in vivo with significant reduction in tumor growth. We have established a model to define this anti-tumor activity and its potential toxic effects through transduction of sarcoma with LV/hu-IL-12 and autologous reintroduction immunotherapy.

## Supplementary Information

Supplementary Figures.

## Data Availability

All data generated or analyzed during this study are included in this published article (and its supplementary information files).

## Abbreviations

AML: Acute myeloid leukemia
AZT: Azidothymidine
CTFR: Cell trace far-red
CAR: Chimeric antigen receptor
CW: Children’s Wisconsin
DMEM: Dulbecco’s modified Eagle’s medium
E:T: Effector:target
eGFP: Enhanced green fluorescent protein
FBS: Fetal bovine serum
fLUC: Firefly luciferase
FCM: Flow cytometry
hu-IL-12: Human IL-12
IRB: Institutional review board
IFN-γ: Interferon-γ
IL-12: Interleukin 12
IL-15: Interleukin 15
LV: Lentiviral
LNGFR: Low-affinity nerve growth factor receptor
MCW: Medical College of Wisconsin
MACC: Midwest Athletes Against Childhood Cancer
MOI: Multiplicity of infection
NK: Natural killer
NSG: NOD scid gamma (NOD.Cg-Prkdc^scid^ Il2rg^tm1Wjl^/SzJ)
NSG.Tg(Hu-IL-15): NOD scid gamma transgenic expressing Human Interleukin 15
PBMC: Peripheral blood mononuclear cell
TCR: T-cell receptor
TMPK: Thymidylate kinase
