# Supplementary material for: Sarcoma IL-12 overexpression facilitates NK cell immunomodulation
Source: Sci Rep. 2021 Apr 15;11:8321. doi: 10.1038/s41598-021-87700-2 (PMC8050085; doi:10.1038/s41598-021-87700-2)
Supplement: Supplementary file 1 — Supplementary Figures. [file 41598_2021_87700_MOESM1_ESM.pdf]

Supplementary Information:

Sarcoma IL-12 overexpression facilitates NK cell immunomodulation:

Authors:

Mary Jo Rademacher BS[1], Anahi Cruz BS[1], Mary Faber PhD[1], Robyn A. A. Oldham BS[1,3], Dandan Wang BS[4,5],

Jeffrey A. Medin PhD. [1,2,3], Nathan J. Schloemer MD[1*]

[1]Departments of Pediatrics,[2]Biochemisty and [4]Microbiology and Immunology; Medical College of Wisconsin, Milwaukee, WI; [3]Department of Medical Biophysics, University of Toronto, Toronto, Ontario, Canada; [5]Laboratory [illegible] Molecular Immunology and Immunotherapy, Blood Research Institute, Versiti, Milwaukee, WI.

*Correspondence addressed to NJS (nschloem@mcw.edu)

NJS ORCID: 0000-0003-0473-7379

a

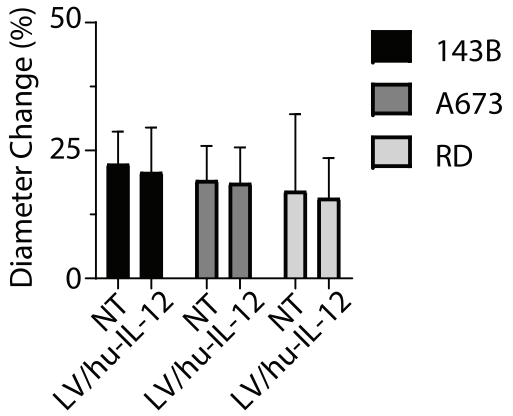

Supplemental Fig. 1 (a) Human sarcomas were plated in ultralow adherent 96-well plates and diameters of the spheroids were recorded at 24 and 48 hours (difference is displayed). N = 16; 2 independent experiments, displayed mean ± standard deviation.

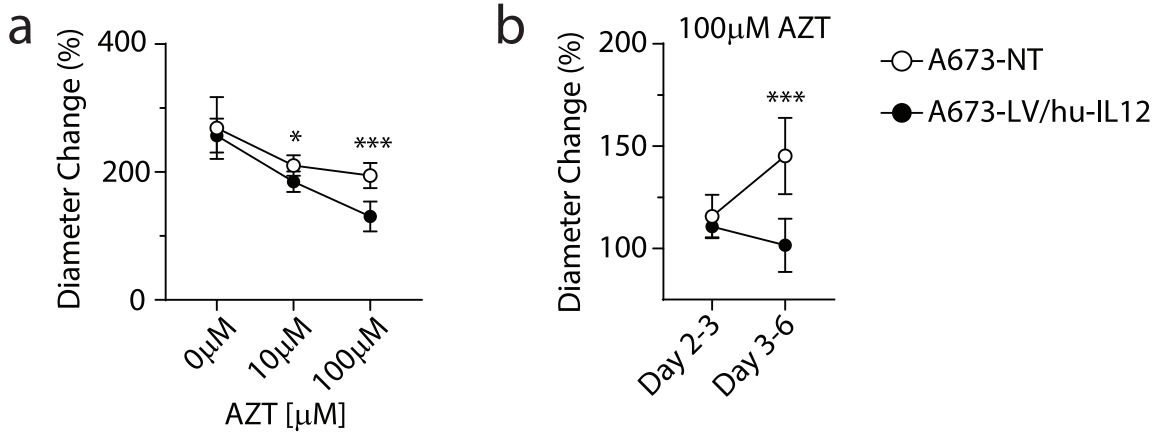

Supplemental Fig. 2 Ewing sarcoma (A673), non-transduced (NT) and LV/hu-IL-12 with TMPK cell-fate control were plated in ultralow adherent 96-well plates. After 24 hours, spheroid formation was verified, diameters were recorded, and AZT was added in escalating dosages. Diameters were recorded for 5 days (2-6), with re-dosing of AZT after 3 days. N = 8; 2 independent experiments, displayed mean ± standard deviation. * = $p < 0.05$, *** = $p < 0.0001$. (a) Displayed in dose escalation response with diameter difference displayed between 24 and 48 hours. (b) Displayed over 5 days of exposure at 100μM AZT.

a

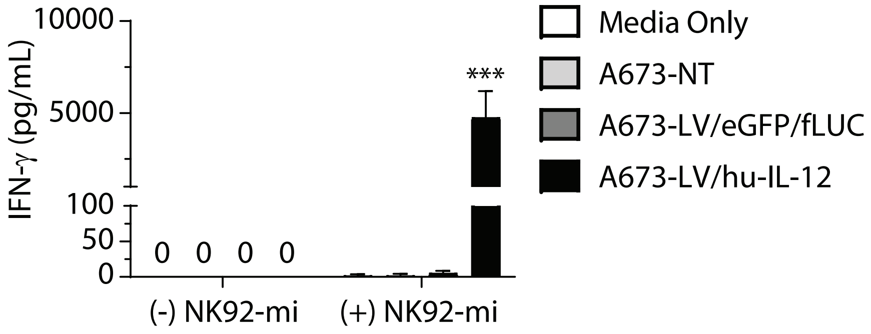

Supplemental Fig. 3 (a) Ewing sarcoma (A673) following no transduction (NT), LV/eGFP/fLUC, or LV/hu-IL-12 transduction were plated in ultralow adherent 96-well plates. After 48 hours, media or NK-92mi at a 10:1 ratio were added. Supernatants assessed by ELISA for IFN-γ. N = 4, displayed mean ± standard deviation. *** = $p < 0.0001$.

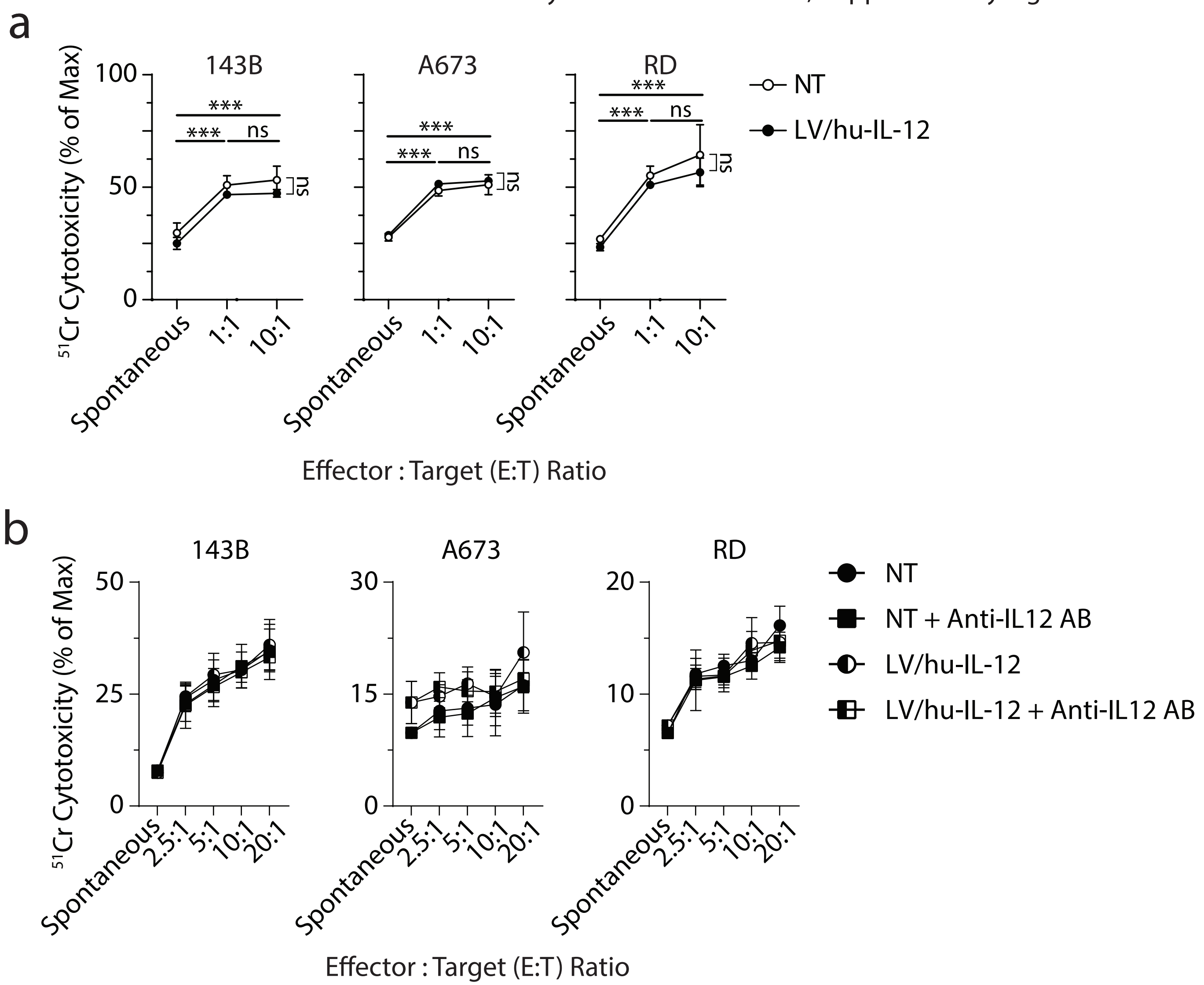

Supplemental Fig. 4 (a) Non-transduced (NT) and LV/hu-IL-12 transduced human sarcoma lines for osteosarcoma (143B), Ewing sarcoma (A673), and rhabdomyosarcoma (RD) were loaded with ^{51}Cr and plated in ultralow adherent 96-well plates. After 24 hours, spheroid formation was verified and NK-92mi cells were added in increasing concentrations. Supernatant was collected and assessed for radioactivity. N = 4, displayed mean ± standard deviation. * = $p < 0.05$, ** = $p < 0.001$, *** = $p < 0.0001$. (b) NT and LV/hu-IL-12 transduced human sarcoma lines were loaded with ^{51}Cr and plated in monolayer with or without presence of 0.5mg/mL of anti-IL-12 blocking antibody. NK-92mi cells were added in increasing concentrations. Supernatant was collected and assessed for radioactivity. N = 6, 2 independent experiments, displayed mean ± standard deviation. Curves are without significant differences.

Mary Jo Rademacher et al., Supplementary Figure 5

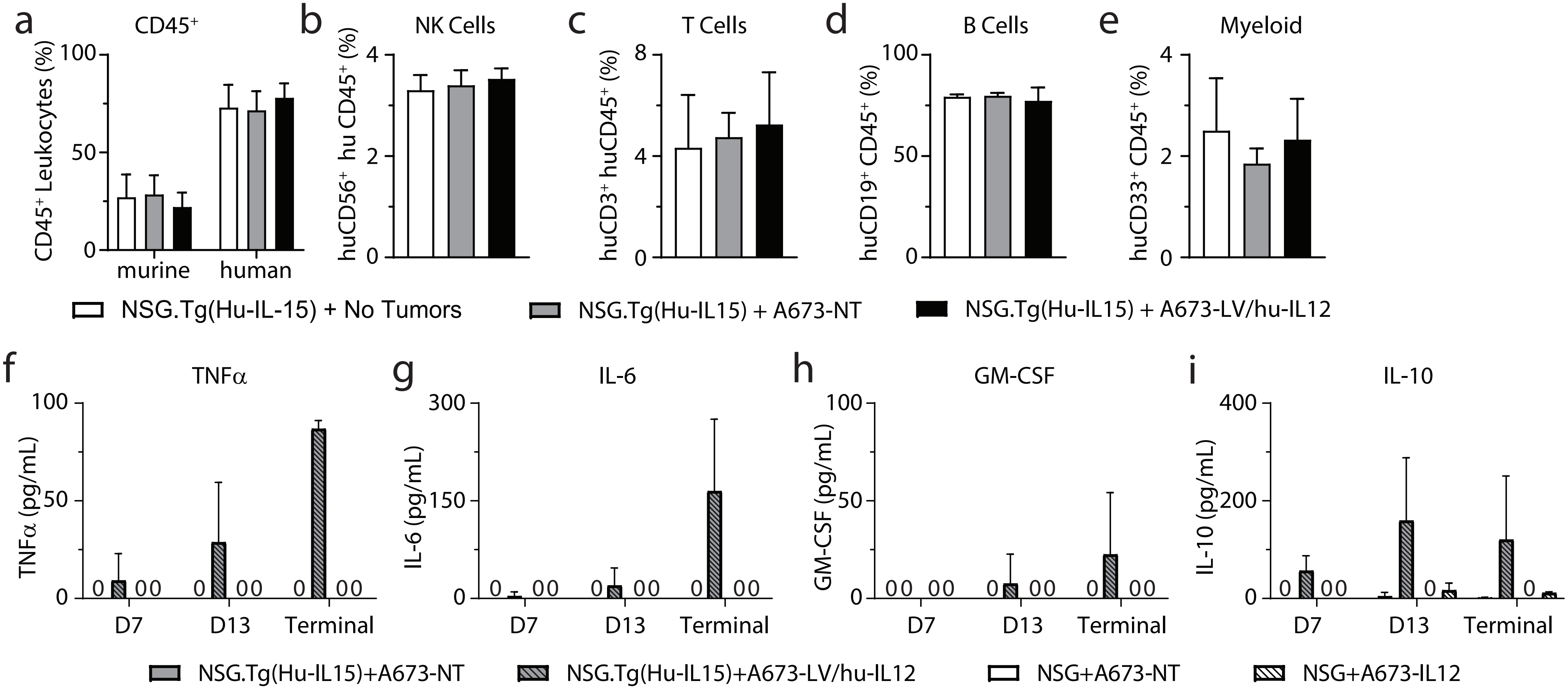

Supplemental Fig. 5 Humanization for NSG.Tg(Hu-IL-15) mice prior to tumor implantation for (a) CD45%, (b) NK cells, (c) T cells, (d) B cells, and (e) myeloid cells. Ewing sarcoma (A673) non-transduced or LV/hu-IL-12 transduced sarcoma line cells were implanted in immunocompromised NSG and NSG.Tg(Hu-IL-15) mice with or without humanization. Serum was collected from peripheral blood prior to injection and weekly until death or at 35 days post tumor injection. Multiplex cytokine analysis performed with display of (f) TNF-α, (g) IL-6, (h) GM-CSF, and (i) IL-10. N=3-4 displayed as mean ± standard deviation.

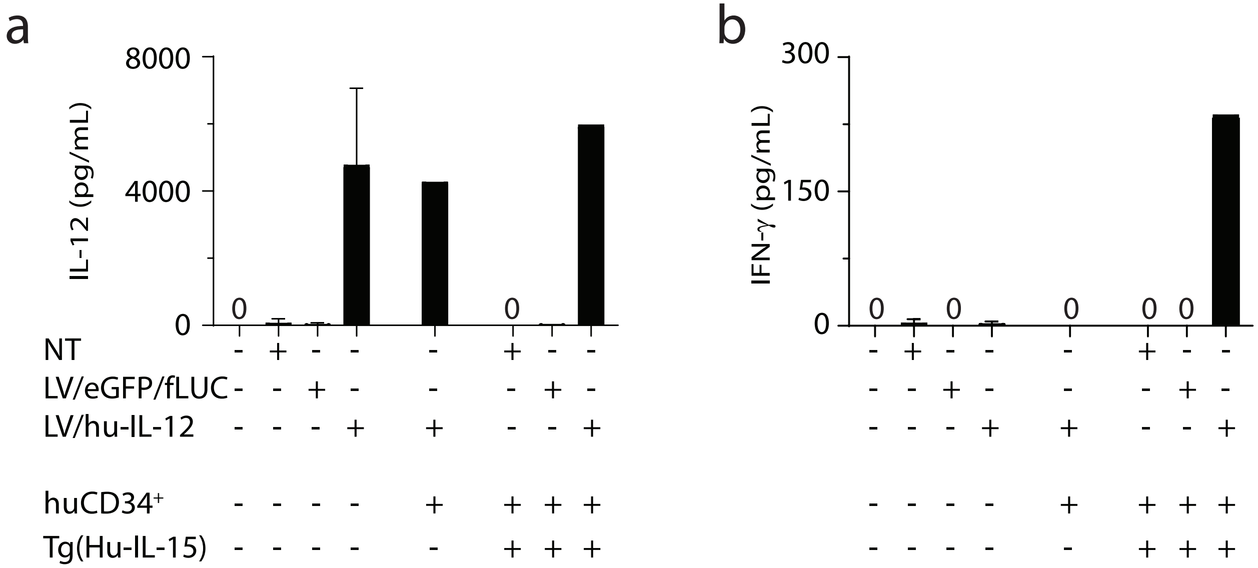

Supplemental Fig. 6 NSG and NSG.Tg(Hu-IL-15) mice were irradiated and transplanted with human CD34+ cells via tail vein injection. Peripheral blood engraftment was confirmed by FCM (human CD34+ >25% of CD34 population) at 6 weeks. Ewing sarcoma (A673) non-transduced, LV/eGFP/fLUC, or LV/hu-IL-12 transduced sarcoma lines were implanted. Serum was collected from peripheral blood with (a) IL-12 and (b) IFN-γ (day 23 or terminal) measured by ELISA. N = 1-3; displayed mean ± standard deviation, no statistical comparison secondary to N.
